# Factors Affecting Telehealth Consultation in Diabetic Patients: A Systematic Scoping Review

**DOI:** 10.1155/ijta/4696370

**Published:** 2026-08-16

**Authors:** Mahsa Fadaie Fini, Tahereh Shafaghat, Mahdi Radabadi, Enayatollah Homaie Rad, Sajjad Bahariniya, Seyyed Reza Darijani, Mohammad Ranjbar

**Affiliations:** ^1^ Health Policy and Management Research Center, Department of Health Management and Economics, School of Public Health, Shahid Sadoughi University of Medical Sciences, Yazd, Iran, ssu.ac.ir; ^2^ Social Determinants of Health Research Center, Trauma Institute, Guilan University of Medical Sciences, Rasht, Iran, gums.ac.ir; ^3^ Department of Healthcare Services Management, School of Health Management and Information Sciences, Iran University of Medical Sciences, Tehran, Iran, iums.ac.ir; ^4^ School of Medicine, Tehran University of Medical Sciences, Tehran, Iran, tums.ac.ir

**Keywords:** barriers, diabetes, diabetes mellitus, facilitators, scoping review, telehealth

## Abstract

**Objectives:**

The global rise in diabetes highlights the need for innovative healthcare solutions. This scoping review is aimed at identifying and categorizing patient‐reported facilitators and barriers influencing the use of telehealth consultations among people living with diabetes.

**Methods:**

A systematic scoping review was conducted following the Joanna Briggs Institute (JBI) methodology and PRISMA‐ScR guidelines.

**Data Sources:**

Four databases—PubMed, Scopus, ISI Web of Science, and Google Scholar—were searched from inception to July 31, 2025.

**Eligibility Criteria:**

Included studies were original research or review articles published in English or Persian that examined patient‐reported facilitators and barriers influencing the use of telehealth among people living with diabetes.

**Data Extraction and Synthesis:**

Two reviewers independently screened titles, abstracts, and full texts. Data were extracted using a predesigned form and synthesized thematically into facilitators and barriers.

**Results:**

Of 1673 identified records, 72 met inclusion criteria. Facilitators were grouped into four themes: process (e.g., time and cost savings), structural (e.g., system usability), communication (e.g., improved access), and attitudinal factors (e.g., awareness). Barriers included infrastructure limitations, digital literacy, privacy concerns, and resistance to change.

**Conclusions:**

Telehealth offers significant potential for diabetes management, but systemic and individual‐level barriers must be addressed. Policymakers and healthcare providers should address patient‐reported barriers while strengthening key facilitators to improve the adoption and sustainability of telehealth services in diabetes care.

## 1. Introduction

Chronic diseases represent a major global public health challenge [1], significantly impairing patients′ quality of life, productivity, and functional well‐being [2]. Among these, diabetes stands out as one of the costliest conditions worldwide due to its chronic nature and associated complications [3]. It is projected to incur economic losses of US$2.1 trillion—equivalent to 2.2% of global GDP—by 2030 [4].

According to the International Diabetes Federation, 463 million people worldwide were living with diabetes in 2019, a number expected to rise to 700 million by 2045 [5]. In Germany, for example, over 9.5 million adults were diagnosed with diabetes in 2019, the vast majority of whom had type 2 diabetes mellitus (T2DM) [6]. T2DM is one of the most prevalent metabolic disorders globally, and its incidence is projected to increase in the coming years. This growing patient population drives heightened demand for healthcare services [7].

Living with diabetes requires lifelong self‐management, including regular blood glucose monitoring, adherence to medication, dietary restrictions, physical activity, and routine medical follow‐up [8, 9]. These daily responsibilities can impose considerable physical, psychological, social, and financial burdens on patients [10, 11]. Many individuals experience stress, anxiety, fear of complications, treatment fatigue, and reduced quality of life [10–12]. In addition, frequent healthcare visits, medication expenses, transportation costs, and time away from work further increase the burden of diabetes [12]. These challenges emphasize the importance of patient‐centered healthcare approaches that improve access to care while minimizing disruption to patients′ daily lives [8–11].

In a 2019 meta‐analysis, Tchero et al. [13] examined the clinical efficacy of telemedicine in diabetes management. Their findings revealed significant reductions in HbA1c levels among patients receiving telehealth support. Notably, individuals with type 2 diabetes, particularly older adults, derived the greatest benefit from these interventions.

The rising number of patients with chronic and multimorbid conditions presents significant challenges to the organization and financing of healthcare systems worldwide. These individuals have complex needs and often transition across multiple care settings, requiring targeted strategies to ensure effective care delivery [14]. A study by Savira et al. [15] in Australia found that telehealth was viewed as an appealing option that eliminated long travel and allowed consultations with a familiar physician. Cost was a major influencing factor, particularly when exceeding $30. Telehealth was least preferred for new or unfamiliar physical symptoms but was more acceptable for postsurgical follow‐ups.

Across healthcare systems, the implementation of telehealth has the potential to enhance care quality and clinical outcomes, reduce mortality and hospital utilization, and complement traditional treatment modalities [16]. Telehealth visits can also minimize exposure to infectious diseases, reduce travel time and costs [17], and improve access to healthcare and specialist services—particularly for marginalized and minority populations [18]. Moreover, telehealth has demonstrated effectiveness in managing chronic conditions and medication adherence [16].

Telehealth—sometimes referred to as telemedicine or teleconsultation in the literature—is a term that has been defined in various ways over the past 4 decades [19]. In this review, the term telehealth consultation is used as an umbrella concept referring to the delivery of healthcare services using information and communication technologies (ICT) to support remote interactions between patients and healthcare providers. This includes synchronous modalities (e.g., telephone and video consultations) and asynchronous communication. Although the terms telemedicine and teleconsultation are often used interchangeably in the literature, they are considered synonymous for the purpose of this study, and the term telehealth consultation is used consistently throughout this review.

Global health services underwent unprecedented transformation during the COVID‐19 pandemic, including the rapid adoption of teleconsultation [20]. In the United States, for example, many healthcare providers shifted to telephone or video appointments to reduce the risk of viral transmission [21]. The COVID‐19 pandemic accelerated the digital transformation of healthcare systems worldwide. In the United Kingdom, healthcare policy has increasingly promoted the digitalization of healthcare services to improve accessibility, convenience, efficiency, and cost‐effectiveness. Although digital transformation initiatives had already been introduced before the pandemic, COVID‐19 substantially accelerated the routine implementation of telehealth services [22, 23].

The adoption rate of telehealth technologies is approximately 60% in high‐income countries, compared with around 20% in low‐ and middle‐income countries. Despite the introduction of a new health insurance system and comprehensive clinical guidelines for online medicine in Japan in 2018, telemedicine has yet to achieve widespread acceptance. One key barrier is the lack of a clear economic advantage. Additionally, strict and specific regulations have eroded the time and geographic flexibility that telemedicine is intended to provide [24].

Between July and December 2022, 58.5% of US adults used the internet to seek health or medical information [25]. According to a survey by the Pew Internet and American Life Project, 41% of patients reported that “the internet influences their decisions about whether to visit a doctor, how to treat an illness, or what questions to ask their physician” [26].

In Australia, several specialist telehealth services have been available since the early 2000s for individuals living in rural and remote areas—including psychiatric services (since 2002), specialist physician consultations (since 2011), allied mental health services (since 2017), and select general practitioner (GP) services (since 2019). However, prior to the COVID‐19 pandemic, telehealth was not routinely accessible for primary care or specialist appointments in urban settings [27].

Despite growing evidence supporting the effectiveness of telehealth in diabetes care, previous reviews have primarily focused on clinical outcomes, implementation strategies, or technology‐specific interventions, with limited attention to the broad range of patient‐reported facilitators and barriers influencing telehealth use. Moreover, existing evidence has not been synthesized within a comprehensive patient‐centered framework that integrates these factors across different domains. Addressing this gap is important because patients′ perspectives are fundamental to the successful implementation and long‐term sustainability of telehealth services. Therefore, this scoping review is aimed at identifying and synthesizing patient‐reported facilitators and barriers influencing the use of telehealth consultations among people living with diabetes.

## 2. Methods

This study was a scoping review conducted in accordance with the Joanna Briggs Institute (JBI) methodology, including both the 2020 protocol [28] and the updated 2024 JBI Manual for Evidence Synthesis [29], and reported using the Preferred Reporting Items for Systematic Reviews and Meta‐Analyses extension for Scoping Reviews (PRISMA‐ScR) checklist [30]. The full methodological framework is outlined below.

*A review protocol was not prospectively registered. Nevertheless, the review was conducted according to the Joanna Briggs Institute* (*JBI*) *methodology and reported in accordance with the PRISMA-ScR guideline to ensure methodological rigor and transparency*.

### 2.1. Selecting the Research Question

In accordance with the JBI guidelines for scoping reviews, the primary research question was formulated as follows:

*What patient-reported facilitators and barriers influence the use of telehealth consultations among people living with diabetes?*

Establishing this question clarifies the alignment between the study′s purpose and its central inquiry. Furthermore, the scoping review question directly informs the development of specific inclusion criteria. A precisely articulated question supports protocol design, enhances the efficiency of the literature search, and provides a coherent framework for reporting the review′s findings.

### 2.2. Searching for Related Studies

In this phase, the researchers conducted a comprehensive search across three major databases—Web of Science, PubMed, and Scopus—using relevant keywords. Google Scholar was searched as a supplementary source to identify potentially relevant studies not indexed in the primary databases. Owing to the large number of records retrieved, screening was limited to the first 200 results ranked by relevance, consistent with common practice in evidence synthesis. The full search strategy is detailed in Table 1.

**Table 1:** Search strategy used in the study.

Databases	ISI Web of Science, PubMed, Scopus, Google Scholar	
Limitations	Language (English/Persian), in title/abstract (keywords), full text available, study type: original or review	
Search timeframe	Database inception to July 31, 2025	
Search time	Initial search: October 29, 2023	
	Updated search: July 31, 2025	
Search terms	#1	Diabetes OR “diabetes mellitus” [mesh]
	#2	Choice∗ OR desire∗ OR preference∗ OR willingness∗ OR option∗ OR alternative∗
	#3	“Telehealth” OR “telemedicine” OR “telecommunication” OR telemedicine [mesh]
Search strategy	#1 AND #2 AND #3	
Search strategy in PubMed	(“Diabetes”[title/abstract] OR “diabetes mellitus”[MeSH terms]) AND (“choice∗”[title/abstract] OR “desire∗”[title/abstract] OR “preference∗”[title/abstract] OR “willingness∗”[title/abstract] OR “option∗”[title/abstract] OR “alternative∗”[title/abstract]) AND (“telehealth”[title/abstract] OR “telemedicine”[title/abstract] OR “telecommunication”[title/abstract] OR “telemedicine”[MeSH terms])	
Search strategy in ISI Web of Science	((TS = (diabetes)) AND TS = (choice∗ OR desire∗ OR preference∗ OR willingness∗ OR option∗ OR alternative∗)) AND TS = (“telehealth” OR “telemedicine” OR “telecommunication”)	
Search strategy in Scopus	(TITLE‐ABS‐KEY (diabetes) AND TITLE‐ABS‐KEY (choiceOR desire∗ OR preference∗ OR willingness∗ OR option∗ OR alternative∗) AND TITLE‐ABS‐KEY (“telehealth” OR “telemedicine” OR “telecommunication”))	

The initial database search was conducted on October 29, 2023, and retrieved studies published from database inception to October 29, 2023. An updated search using the same strategy was conducted on July 31, 2025, to identify studies published between October 30, 2023, and July 31, 2025. All eligible studies identified in the updated search were incorporated into the final review. The search strategy remained unchanged. All additional studies meeting the inclusion criteria were incorporated into the final analysis.

*To maximize the sensitivity of the search, the search strategy included preference-related terms* (*e*.*g*., *preference*, *choice*, *willingness*, *and desire*), *as studies reporting patient-reported facilitators and barriers to telehealth use often describe these factors using preference-related terminology*.

### 2.3. Studies Selection

#### 2.3.1. Eligibility Criteria (PCC Framework)

• Population: adults (≥ 18 years) who are diagnosed with T2DM.
• Concept: preferences (Patient‐reported facilitators and barriers) influencing the use of telehealth consultations. The concept of interest included any patient‐reported factor (e.g., facilitator, barrier, enabler, challenge, or system characteristic) that influenced the use, adoption, uptake, or continued engagement with telehealth services for diabetes care. Studies focusing on patient experiences, perceptions, and reported determinants of telehealth use were eligible, irrespective of whether these factors were described as facilitators or barriers.
• Context: any healthcare setting—primary, secondary, or community care—globally.

Included studies were original research or review articles published in English or Persian with full text available that explicitly examined patient‐reported facilitators and barriers influencing the use of telehealth among people living with diabetes. Eligible study designs included quantitative, qualitative, mixed‐methods, and narrative or systematic reviews reporting empirical or synthesized evidence on the topic. Nonresearch formats (e.g., editorials and commentaries) and nonfull‐text articles were excluded

During this phase, efforts were made to identify relevant gray literature or studies potentially missed during the initial search by reviewing reference lists of included articles and, where feasible, contacting experts or corresponding authors. Backward citation tracking was performed by screening the reference lists of all included studies. Forward citation tracking was also undertaken using Google Scholar to identify newer studies citing the included articles.

### 2.4. Data Extraction

Following the systematic search across all databases and removal of duplicates, two independent reviewers from the research team screened the studies in three sequential phases: title, abstract, and full‐text review. Data from the final included studies were then extracted by the same two reviewers. At each stage, inclusion decisions were based on consensus; in cases of disagreement, a third reviewer provided arbitration.

EndNote software (Version 21.2) was used to manage the search and deduplication process. A standardized data extraction form was developed in Microsoft Excel to capture key study characteristics aligned with the research question, including author(s), publication year, country, study title, study design, and contextual details. The PRISMA‐ScR [30] guided the evidence selection process, and a PRISMA flow diagram [31, 32] was used to visually represent the study screening and selection workflow.

In accordance with current JBI guidance for scoping reviews [28, 29, 33], formal critical appraisal of individual studies was not performed, as the primary objective of this review was to map the breadth and nature of existing evidence on patient preferences (facilitators and barriers)—not to evaluate the methodological quality of the included studies.

### 2.5. Data Synthesis

Data synthesis followed a thematic analysis approach. Two reviewers (M.F.F. and T.S.) independently extracted relevant data (e.g., reported facilitators, barriers, and influencing factors) from the included studies into a predesigned data charting form in Microsoft Excel.

The synthesis process was iterative and inductive. Initially, the reviewers familiarized themselves with the data and generated initial descriptive codes capturing specific factors mentioned in the studies. These codes were then discussed and grouped based on conceptual similarities to form preliminary subthemes.

*For example*, *patient statements describing reduced travel time and shorter waiting times were initially coded as* “*time saving*,” *whereas statements describing reduced transportation or consultation expenses were coded as* “*cost saving*.” *These initial codes were then grouped into the subthemes* “*Save time*” *and* “*Save cost*,” *which were subsequently incorporated into the overarching theme* “*Process factors*.” *Similarly*, *codes related to user-friendly interfaces*, *system accessibility*, *and integration with electronic health records were grouped under the subtheme* “*Online*/*virtual system capabilities*,” *which formed part of the broader theme* “*Structural factors*.”

Through repeated discussions and refinement between the reviewers, these subthemes were further analyzed and clustered into broader, overarching themes that represented the core domains of factors influencing patient preferences (facilitators and barriers). Coding was performed independently by two reviewers throughout the analysis. Discrepancies in coding or thematic grouping were resolved through iterative discussion and consensus. When consensus could not be reached, a third senior reviewer (M.R.) reviewed the data and made the final decision. Coding was conducted independently by the two reviewers throughout the analysis. Rather than calculating formal interrater agreement statistics, discrepancies were resolved through iterative discussion and consensus, consistent with qualitative thematic synthesis approaches recommended for scoping reviews. Formal interrater reliability statistics (e.g., Cohen′s kappa) were not calculated because the thematic synthesis followed an iterative consensus‐based approach consistent with JBI guidance for scoping reviews.

This process culminated in the development of the final analytical framework consisting of four primary themes: (1) process factors, (2) structural factors, (3) communication factors, and (4) attitudinal factors. Each primary theme contained several distinct subthemes. All extracted data from the 72 included studies were then mapped onto this final framework, which is presented comprehensively in Tables 2 (facilitators) and 3 (barriers). This descriptive analytical approach aligns with the JBI methodology for scoping reviews, which focuses on mapping the key concepts and evidence in a field [33].

*Because some factors were conceptually related across domains*, *theme allocation was based on the primary conceptual function of each factor rather than exclusive categorization*. *For example*, *system usability and flexibility were classified under structural factors when they referred to technical or platform-related characteristics*, *whereas ease of communication was classified under communication factors when it reflected patients*′ *interactions with healthcare providers or the perceived quality of communication*. *This approach ensured conceptual consistency while acknowledging the interrelated nature of some telehealth determinants*.

**Table 2:** Facilitators for using telehealth services in diabetic patients.

Themes	Subthemes	Qualitative codes
Process factors	Save time	Reduced waiting time for receiving services and timely responses [17, 24, 34–46]
		Shorter examination times [17, 34, 35, 47–55]
		No need to visit healthcare facilities [4, 24, 35, 37, 38, 51, 56–58]
		Improved time efficiency for recording and electronically transferring patients′ health data (e.g., blood glucose readings) to healthcare providers [6, 7]
	Save cost	Being free or affordable [17, 24, 34–37, 39, 41, 46, 47, 53–55, 59–68]

Communication factors	Ease of use and improved communication	Greater adherence to virtual appointments compared with in‐person visits [34, 59]
		Improved access to healthcare regardless of geographical location and greater convenience in utilizing virtual services [38, 43, 45–47, 49, 52, 55, 59, 69–76]
		Access to the internet, subsidies, smartphones, or devices such as glucometers [3, 4, 7, 37, 48, 77]
		Receiving medication and care at any time and from any location [4, 34, 35, 41, 44]
		More time available for family responsibilities and professional duties [17, 40, 55]
		Improved communication between patients and providers, with adequate time to consult with the doctor [6, 7, 37, 45–47, 50, 61, 70, 78–80]
		Enhanced peer support through telehealth platforms, enabling communication and information sharing among patients [6, 7, 37, 61, 67, 71, 74, 79, 81]
		Ability to effectively express concerns and clearly explain health issues [50, 78]
		Patient engagement in treatment and autonomy in selecting consultation modality [2, 35]

Structural factors	Online/virtual system capabilities	User‐friendliness of virtual applications [54, 61, 70, 71, 82, 83]
		Clarity of auditory and visual features [36, 50, 52, 59, 78]
		Availability of the application in the native language [49]
		Simplicity and comprehensibility of the system [6, 34, 50, 61]
		Flexibility of the system [84]
		Use of mobile phones as reminders for appointments [56, 74]
		Availability of secure backups of patients′ health records and telehealth data [34]
		Enhancement of content within virtual applications [7]
		Ability to integrate data with electronic health records [34, 71, 82]
		Customized feedback and personalization of virtual applications [74]
		Receiving tangible rewards, such as gift cards, cash, reduced health insurance premiums, or intangible rewards like badges and points [74]
		Boosting motivation through entertaining features of the program [1]
		Interest in virtual applications and willingness to use them in the future [50, 78]
		Technical support, with interest in tracking, follow‐ups, and the presence of a support team [36, 59, 74, 84–86]
		Receiving advice and guidance from providers [81]
	Privacy and confidentiality	Ensuring security, privacy, and patient safety [1, 34, 60, 61, 69]
		Guaranteeing data confidentiality, protection against breaches, and safeguarding personal information [34, 68, 69, 78]
		Utilizing anonymized data for research purposes [60]
		Ability to lock mobile devices using passwords [56]
		Establishing trust in virtual applications [39, 72, 86]

Attitudinal factors	Patient education and awareness	Providing educational information to patients, addressing their educational needs, and explaining their condition [1, 6, 7, 20, 33, 49, 61, 74, 75, 79, 81, 84, 87]
		Patients′ digital literacy and competency [62, 77, 87]
		Healthcare providers′ knowledge, digital skills, and competence in delivering telehealth services [33]
		Understanding and acceptance of technology [72, 77, 78, 86]
		Perceived experiences and personal beliefs regarding technology [61, 78]
	Quality and efficiency of service delivery	Improved service quality [42, 43, 59, 66, 67, 83, 87]
		Continuity of care [20]
		Early detection capabilities [66, 75]
		Meeting patients′ healthcare needs [50, 59]
		Satisfaction with service quality and outcomes [39, 43, 50, 59, 68, 78, 83]
		Promoting independence and self‐management for patients [1, 40, 60, 61, 85]
		Increasing efficiency, effectiveness, and productivity [4, 17, 39, 43, 53, 69, 72, 76, 83]
		Improvement in health outcomes [43, 53, 60, 83]
		Lifestyle modifications and better understanding of one′s condition [1, 37]
		Reducing anxiety and fostering reassurance and calmness [1, 51]
		Higher adherence to treatment protocols [2]
		Encouraging physical activity [1]
		Providing integrated services (combination of virtual and in‐person services) [73]
		Service delivery through video, email, SMS, or audio (e.g., preference for visual content over audio) [34, 84]

**Table 3:** Barriers for using telehealth services in diabetic patients.

Themes	Subthemes	Qualitative codes
Process factors	Lack of time	Short duration of virtual visits [54, 76]
	Lack of facilities	Equipment and facilities [17, 82, 88]
		Limited access to computers, smartphones, or even internet [35, 43, 49, 63, 71, 74, 76, 82, 88, 89]
		Slow internet connection [17, 34, 80]
	Cost increase	High cost and expense of virtual visits [42, 61, 75, 80, 89]
		High costs of accessing virtual care and lack of funding [75, 90]
		Inadequate reimbursement and insurance coverage [68, 75, 91]

Communication factors	Difficulty communicating and using	Lack of support for the native language [34, 47, 54, 80, 92]
		Limited compatibility and inflexibility of the system [46, 47]
		Absence of alarm notifications [34]
		Misalignment of virtual programs with patient needs [81]
	Lack of social support	Lack of family and peer support [71, 88]
		Absence of alarm notifications [34]
		Misalignment of virtual programs with patient needs [81]
		Small and unclear text in virtual programs [75]

Structural factors	Lack of infrastructure	Poor organizational structure [34, 90]
		Lack of responsiveness to patients and their fear of follow‐up neglect [34, 51, 74]
		Perception of less attention from providers during virtual visits and the provider not requesting the patient to use the application [38, 74]
		Difficulty in speaking and asking questions during virtual visits [38, 53]
		Difficulty of using smartphones and smartwatches and their complexity [63, 64, 75]
		Easier communication in in‐person visits [51, 57]
		Forgetting, delaying, or missing appointment times [46, 75]
		Lack of familiarity with using digital technologies [1]
	Technical support and regulatory issues	Need for support in individuals with specific characteristics, such as hearing impairment [35, 61]
		Lack of integration with existing systems [91]
		Lack of technical support [91, 92]
		Regulatory challenges [46]
		Additional workload for staff/workflows challenges [46, 91]

Attitudinal factors	Concerns	Concerning privacy, security, and data protection [42, 53, 63, 64, 68, 75, 80]
		Lack of trust and confidence in technology [34, 51, 86]
		Concerns about unintended disclosure of patients′ health conditions to unauthorized individuals [63]
		Worries about making errors in self‐care due to perceived difficulties without a physical examination [38]
		In‐person visits being perceived as more personal [57]
		Doubt about the reduction in time and cost‐effectiveness of virtual visits [18]
	Lack of awareness	Lack of awareness and knowledge about the existence of technology [74, 81, 88, 89]
		Low digital literacy and understanding among patients [1, 17, 34, 41, 44, 72, 74, 80–82, 92]
		Resistance to change and reluctance to adopt new skills [1, 75, 86]
		Insufficient knowledge of virtual programs [47, 82]
	Lack of desire and interest	Lack of willingness, interest, and need to use virtual programs [1, 34, 75, 76, 89]
		Rejection of digital technologies [18]
		Preference for in‐person consultations [1, 82]
		Resistance to change [82]
	Low quality	Weakness in remote consultations [88]
		Feeling of greater focus and responsibility from providers during in‐person visits [36, 57, 68]
		Low quality of care or lack of access to quality care [41, 51]
		Insufficient provider knowledge of virtual programs [47]

## 3. Results

**Figure 1:**
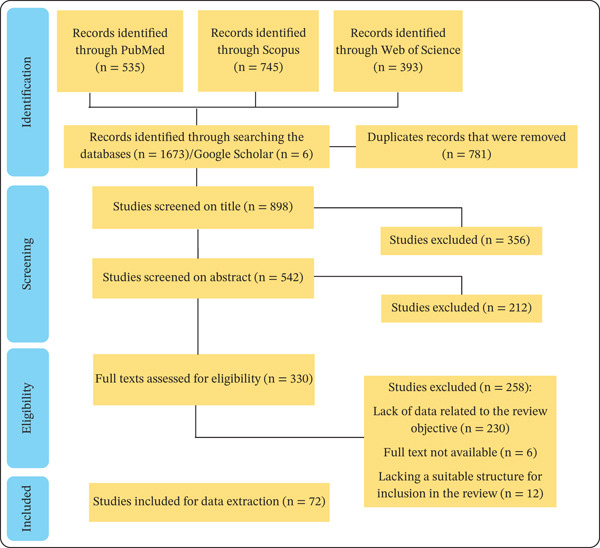
PRISMA flowchart for study selection in the scoping review.

Based on a systematic search, 1673 records were identified across three databases, with an additional six records identified through Google Scholar. After removing 781 duplicate records, 898 studies remained for title screening. Following title screening, 542 studies proceeded to abstract screening. After abstract screening, 330 full‐text articles were assessed for eligibility. Of these, 258 articles were excluded, leaving 72 studies that met the inclusion criteria and were included in the review (Figure 1).

The included studies comprised a range of methodological designs, including qualitative, quantitative, mixed‐methods, cross‐sectional, cohort, randomized, and review studies, reflecting considerable methodological heterogeneity across the evidence base. Over half of the studies (*n* = 48) were published between 2020 and 2025, reflecting the surge in research interest in telehealth for diabetes management—largely accelerated by the COVID‐19 pandemic. Geographically, the studies spanned a wide range of healthcare contexts: the largest proportions were conducted in the United States (*n* = 24) and across various Asian countries (*n* = 17), followed by European nations (*n* = 12), African countries (*n* = 7), and Australia (*n* = 5). This global representation underscores the broad relevance of telehealth across diverse economic and cultural settings. Methodologically, the studies employed a variety of research designs, including quantitative (*n* = 41), qualitative (*n* = 20), mixed methods (*n* = 7), and review articles (*n* = 4), enabling both statistical trend analysis and in‐depth exploration of patient experiences. The target populations predominantly consisted of adults with type 2 diabetes. The telehealth interventions examined were comprehensive and multifaceted, encompassing real‐time video consultations, telephone visits, mobile health (mHealth) applications for self‐management, remote patient monitoring (RPM) systems—particularly for glucose tracking—and specialized services such as teleophthalmology for diabetic retinopathy screening. This breadth captures the full spectrum of technology‐enabled diabetes care (Supporting Table).

**Figure 2:**
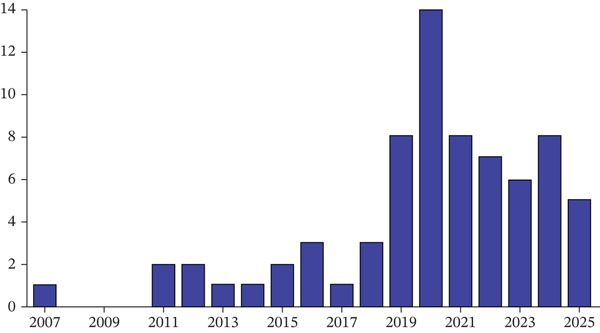
Frequency distribution of included studies by year of publication.

As shown in Figure 2, the number of studies examining facilitators and barriers to telehealth use in diabetes management rose sharply from 2019—the onset of the COVID‐19 pandemic. This surge peaked in 2020, the first full year of the pandemic. Although the volume of studies declined slightly in subsequent years compared with 2020, the overall trend remained upward and substantially higher than levels observed in the preceding decade.

Based on thematic synthesis, facilitators and barriers for using telehealth services in diabetic patients were categorized under four core domains, each with multiple subthemes. Below, we present a narrative summary of the breadth and distribution of them, and full thematic mappings are provided in Tables 2 (facilitators) and 3 (barriers).

### 3.1. Facilitators for Using Telehealth Services in Diabetic Patients

− *Process factors*: Time and cost savings were among the most frequently reported facilitators. Saving time was cited in 27 studies, including reduced travel, waiting, and consultation times [17, 24, 34–45, 47–58, 93]. Cost savings were reported in 22 studies and mainly reflected the affordability of telehealth services, particularly when services were free or offered at lower cost, as well as reduced travel‐related expenses where applicable [17, 24, 35–37, 39, 41, 47, 53–55, 59–68, 93].
− *Structural factors*: User‐friendly interfaces, telehealth systems with flexible, user‐centered features (e.g., simple and easy‐to‐use applications, native‐language availability, appointment reminders, personalized feedback, integration with electronic health records, and access to technical support), together with data privacy and confidentiality, were emphasized [1, 6, 34, 36, 50, 54, 61, 69–71, 78, 82, 83, 85, 86].
− *Communication factors*: Improved access to healthcare regardless of location, greater convenience in using virtual services, and enhanced patient–provider interaction have been reported in 35 studies [34, 38, 43, 45, 47, 49, 52, 55, 59, 69–81, 93].
− *Attitudinal factors*: Awareness, digital literacy, perceived service quality, and treatment adherence were noted in more than 27 studies [1, 6, 7, 20, 35, 39–43, 50, 53, 59–61, 66–68, 72, 74, 75, 78, 79, 81, 83, 84, 87].

### 3.2. Barriers for Using Telehealth Services in Diabetic Patients

− *Process factors*: High costs related to virtual consultations, accessing telehealth services, insufficient funding, and inadequate reimbursement or insurance coverage (*n* = 8 studies) [42, 61, 68, 75, 80, 89–91], lack of time due to short visits (*n* = 2) [54, 76], and inadequate access to devices or internet (*n* = 13) were common [17, 34, 35, 43, 49, 63, 71, 74, 76, 80, 82, 88, 89].
− *Structural factors*: Poor infrastructure, limited system compatibility, inflexible telehealth systems that were difficult to use or insufficiently adapted to patients′ needs, together with insufficient technical support, appeared in 19 studies [1, 17, 18, 34, 35, 46, 47, 51, 61, 63, 64, 74, 75, 82, 88, 90–93].
− *Communication factors*: Language incompatibility, difficulty expressing concerns, and lack of social support were reported in 11 studies [34, 38, 47, 51, 54, 57, 71, 75, 81, 88, 92].
− *Attitudinal factors*: Privacy concerns (*n* = 11) [34, 38, 42, 51, 53, 63, 64, 68, 75, 80, 86], low digital literacy (*n* = 13) [1, 17, 34, 41, 44, 72, 74, 80–82, 88, 89, 92], resistance to change (*n* = 3) [1, 75, 86], and preference for in‐person care (*n* = 6) [36, 41, 51, 57, 68, 88] were recurrent themes.

## 4. Discussion

In this study, we present a comprehensive analysis of all factors influencing telehealth consultations for patients with diabetes, systematically categorized into four core dimensions: process factors, structural factors, communication factors, and attitudinal factors. The primary contribution of this review lies in its novel, patient‐centered framework that integrates both well‐established and underexplored telehealth determinants into four interconnected thematic domains.

The included studies employed diverse methodological approaches, including qualitative, quantitative, and mixed‐methods designs, each contributing complementary insights into patient‐reported facilitators and barriers. Quantitative studies primarily described the frequency or prevalence of reported factors, whereas qualitative studies provided a deeper understanding of patients′ experiences, perceptions, and contextual influences underlying telehealth use. Despite this methodological diversity, considerable convergence was observed across study designs, with recurring themes related to process, structural, communication, and attitudinal factors. This consistency across different methodological approaches strengthens confidence in the robustness of the thematic framework identified in this review.

Previous studies have approached these factors through narrower lenses. For instance, Rogers et al. [47] classified facilitators and barriers to implementing the Mobile Insulin Titration Intervention (MITI) into four categories: characteristics of the intervention, internal organizational context, implementation processes, and attitudes/beliefs of patients and providers. Similarly, Sun et al. [17] grouped barriers and facilitators for sustaining telehealth‐based continuity of care in type 2 diabetes into three themes: perceived benefits of remote visits, perceived challenges, and impacts on care quality.

Although many facilitators and barriers identified in this review are common across chronic diseases, diabetes presents unique management demands because patients require continuous self‐monitoring, frequent treatment adjustments, regular screening for complications, and sustained interaction with healthcare professionals. Therefore, barriers to telehealth may have greater consequences for continuity of care and long‐term disease control in this population. Diabetes is a lifelong condition that requires continuous self‐management, including regular blood glucose monitoring, medication adjustment, dietary management, physical activity, and frequent interaction with healthcare professionals. Consequently, factors that improve access to care, reduce the burden of routine follow‐up, facilitate ongoing communication, and support self‐management may have a particularly important role in diabetes care. Although numerous studies have explored facilitators and barriers to telehealth in diabetes care, most have addressed only isolated dimensions—focusing on technology, cost, or user attitudes—without offering a unified, holistic framework [1, 39–41].

In the sections that follow, we examine the key dimensions influencing telehealth adoption among diabetic patients, organized into two overarching categories: facilitators and barriers, each mapped across the four thematic domains to provide a structured, actionable understanding of patient‐centered telehealth engagement.

### 4.1. Facilitators for Using Telehealth Services in Diabetic Patients

In this study, facilitators influencing telehealth adoption among diabetic patients were systematically categorized into seven subthemes: “save time,” “save cost,” “online/virtual system capabilities,” “privacy and confidentiality,” “ease of use and improved communication,” “patient education and awareness,” and “quality and efficiency of service delivery.” These subthemes were further organized under four overarching conceptual themes: process, structural, communication, and attitudinal factors.

This integrated framework builds upon—but significantly expands—prior taxonomies. For instance, Rogers et al. [47] identified facilitators across four domains (intervention characteristics, internal setting, implementation process, and attitudes/beliefs), yielding 14 subthemes specific to the MITI. Although insightful, that model was context‐bound to a single clinical program. Similarly, Peng et al. [74] highlighted perceived facilitators among rural adults with type 2 diabetes, such as freely accessible mobile applications, tangible rewards (e.g., gift cards), and anonymous incentives to boost engagement. These findings align with our subtheme “online/virtual system capabilities,” particularly regarding motivational design and accessibility.

Zaman et al. [1], in their SWOT analysis of ICT use among older adults with chronic conditions, identified benefits including nonpharmacological support, health education, promotion of physical activity and healthy diets, and enhanced motivation through enjoyable interfacing, all of which contributed to reduced anxiety and improved self‐management. These outcomes map directly onto our attitudinal subthemes: “patient education and awareness” and “quality and efficiency of service delivery,” particularly regarding psychological reassurance and behavioral activation.

Variations in how facilitators are categorized across studies can be attributed to differences in scope, population, technological focus, and theoretical orientation. Some studies prioritize system design; others emphasize user psychology or economic incentives. However, a critical gap remains: Few studies have attempted integrative patient‐centered frameworks. This review addresses that gap by synthesizing evidence into a unified framework that captures the full spectrum of patient‐relevant enablers—spanning logistical, technological, communicative, and psychological dimensions.

#### 4.1.1. Process Factors

Under the “process factors” theme, two subthemes emerged: “save time” and “save cost.” These were among the most consistently reported facilitators across the global evidence base. Time savings for patients, including reduced travel time, waiting time, and time spent attending healthcare appointments, were documented in over 30 studies spanning diverse geographic and socioeconomic contexts [4, 6, 7, 17, 24, 34–58]. Similarly, cost savings for patients were highlighted as pivotal, particularly in settings where telehealth services were offered at low or no cost, reducing patients′ out‐of‐pocket expenses and travel‐related costs [17, 24, 34–37, 39, 41, 46, 47, 53–55, 59–68]. Although Xu et al. [59] provided a compelling quantitative estimate showing an average saving of 78 min and $72.94 per visit, this finding is representative of a much broader pattern observed across literature. The consistency of these reports underscores time and cost efficiency not as isolated benefits, but as foundational enablers of telehealth adoption in diabetes care. These benefits are especially important for people living with diabetes because routine diabetes care requires regular clinic visits for glycaemia assessment, medication review, screening for diabetes‐related complications, and ongoing self‐management support. Reducing travel time and out‐of‐pocket expenses may therefore lessen the long‐term treatment burden experienced by patients and facilitate sustained engagement with diabetes care. The importance of these process‐related facilitators appeared to vary across healthcare settings. In high‐income countries, convenience, flexibility, and reduced travel time were commonly emphasized, whereas studies conducted in low‐ and middle‐income settings more frequently highlighted affordability and reduced transportation costs as key determinants of telehealth use. These findings suggest that the relative importance of individual facilitators is influenced by healthcare infrastructure and socioeconomic context rather than representing universally equivalent priorities.

#### 4.1.2. Structural Factors

Within the “structural factors” theme, two subthemes were identified: “online/virtual system capabilities” and “privacy and confidentiality.” User‐friendly design, multilingual support, visual clarity, and seamless integration with electronic health records were emphasized in over 12 studies as critical to usability and sustained engagement [34, 36, 50, 52, 54, 59, 61, 70, 71, 78, 82, 83]. Notably, features such as personalized feedback, reminders, and gamified incentives (e.g., badges and rewards) were reported to enhance motivation and adherence [1, 74]. Concerning privacy and confidentiality, 12 studies explicitly identified data security, trust in platforms, and assurance of anonymity as nonnegotiable prerequisites for participation [1, 34, 39, 56, 60, 61, 68, 69, 72, 78, 86]. Altabtabaei et al. [34] reported that both patients and providers advocated for platforms as intuitive as social media apps, with robust safeguards against data breaches. These findings confirm that technical excellence must be paired with ethical and legal assurances to foster trust. For people living with diabetes, who frequently upload glucose readings, medication records, and other personal health information, reliable and easy‐to‐use telehealth platforms are particularly important for supporting long‐term disease monitoring and sustained engagement with care.

#### 4.1.3. Communication Factors

Under this theme, the subtheme “ease of use and improved communication” emerged as a key facilitator. Enhanced access, convenience, and perceived quality of interaction were reported in more than 23 studies [3, 4, 7, 37, 38, 43, 45–49, 52, 55, 59, 69–77]. Patients valued the ability to consult from home, avoid travel, and allocate saved time to family or work responsibilities [4, 34, 35, 41, 44]. For people living with diabetes, maintaining regular communication with healthcare professionals is particularly important because treatment decisions often depend on blood glucose trends, medication adherence, dietary habits, and lifestyle modifications. Telehealth therefore supports the continuous interactions required for effective diabetes self‐management. Moreover, many noted that virtual visits allowed more focused, less rushed conversations with providers [6, 7, 37, 45–47, 50, 61, 70, 78–80]. Fung et al. [50] found that 72% of participants expressed interest in continuing remote care, citing high usability of both telephone and video modalities. However, this preference was not isolated; as results show, similar sentiments were echoed across North America, Asia, Europe, and Australia, reflecting a global consensus on the communicative value of well‐designed telehealth services.

#### 4.1.4. Attitudinal Factors

This theme encompassed two subthemes: “patient education and awareness” and “quality and efficiency of service delivery.” Digital literacy, disease understanding, and perceived usefulness of technology were identified in nearly 18 studies as key drivers of acceptance [1, 6, 7, 20, 33, 49, 61, 62, 72, 74, 75, 77–79, 81, 84, 86, 87]. Educational content embedded within apps or consultations was consistently linked to improved self‐management and confidence [1, 37, 74]. This finding is particularly relevant for diabetes, where successful disease management relies heavily on patients′ knowledge, self‐efficacy, and ability to make informed day‐to‐day decisions regarding glucose monitoring, medication use, nutrition, and physical activity. Consequently, improving digital literacy and patient education may directly enhance diabetes self‐management. Regarding service quality, 19 studies associated telehealth with higher satisfaction, continuity of care, early detection, and better health outcomes [4, 17, 20, 39, 42, 43, 50, 53, 59, 66–69, 72, 75, 76, 78, 83, 87]. Chisolm et al. [76] noted that adolescents engaged most with platforms they found personally relevant and easy to use, finding echoed across age groups in our synthesis. Importantly, psychological benefits such as reduced anxiety and increased reassurance were recurrent themes [1, 51], reinforcing that telehealth′s value extends beyond clinical metrics to emotional well‐being.

Although this review focuses on factors directly reported by patients, it is critical to acknowledge the broader systemic context. The surge in telehealth adoption during and after the COVID‐19 pandemic was largely enabled by temporary policy relaxations—particularly regarding reimbursement, cross‐state licensure, and privacy regulations [94, 95]. Although patients may not explicitly cite these policy shifts as personal facilitators, they represent foundational enablers that render telehealth services accessible, available, and economically sustainable. Patient‐reported benefits such as cost savings and improved access are often downstream effects of these upstream structural changes. Therefore, to ensure the long‐term viability and scalability of telehealth, policymakers and healthcare leaders must recognize supportive, permanent regulatory frameworks as indispensable, systems‐level facilitators.

Although several facilitators were consistently reported across studies, important variations and apparent contradictions were also observed. For example, while most studies described telehealth as improving convenience, accessibility, and communication, others reported that some patients perceived virtual consultations as less personal or insufficient for addressing complex health concerns. Similarly, although telehealth frequently reduced travel‐related costs, financial barriers remained important in settings where reimbursement policies were limited or patients were required to bear technology‐related expenses. These contrasting findings suggest that the effectiveness and acceptability of telehealth are highly context‐dependent and influenced by healthcare systems, patient characteristics, and local implementation strategies.

### 4.2. Barriers to Using Telehealth Services in Diabetic Patients

Barriers were systematically mapped onto the same four thematic domains process factors, structural factors, communication factors, and attitudinal factors; yielding 11 subthemes. This structure enables a nuanced understanding of how logistical, technological, relational, and psychological obstacles intersect.

Previous studies have examined barriers through narrower or context‐specific lenses. For example, Rogers et al. [47] identified three main barrier themes related to implementing the MITI: characteristics of the intervention, internal organizational setting, and staff attitudes and beliefs—yielding six specific subthemes. Although valuable, this framework focused primarily on implementation within clinical workflows rather than patient‐centered experiences.

Similarly, Zaman et al. [1] highlighted barriers among older adults using ICT for chronic disease management, including knowledge gaps, resistance to acquiring new digital skills, and general unwillingness to engage with technology. These align closely with our attitudinal subthemes “lack of awareness” and “lack of desire and interest.” Sipsma et al. [96] explored barriers to adopting smart insulin pens in clinical practice, identifying multilevel challenges: patient‐related (e.g., insurance coverage, cost, smartphone access, training needs), provider‐related, and clinic‐level operational constraints tied to knowledge, capacity, and resources. These findings map onto our structural and process‐related subthemes, such as “cost increase,” “lack of facilities,” and “lack of infrastructure.”

Despite these valuable contributions, few studies have attempted integrative patient‐centered frameworks. Variations in classification likely stem from differences in study scope, population focus, technological context, and theoretical orientation. This review addresses that gap by synthesizing global evidence into a unified, granular framework that captures the full spectrum of patient‐reported and system‐level barriers—providing a foundation for targeted interventions and policy development.

#### 4.2.1. Process Factors

Under this theme, three subthemes emerged: “lack of facilities,” “cost increase,” and “lack of time.” Limited access to smartphones, computers, or reliable internet was reported in 13 studies, particularly in low‐resource and rural settings [17, 34, 35, 43, 49, 63, 71, 74, 76, 80, 82, 88, 89]. High out‐of‐pocket costs and inadequate insurance reimbursement were cited in eight studies as deterrents, especially when telehealth was perceived as more expensive than in‐person care [42, 61, 68, 75, 80, 89–91]. Additionally, some patients felt virtual visits were too brief to address complex needs [54, 76], though this concern was less common than facilitators related to time savings. These barriers may have a greater impact on people living with diabetes because the disease requires lifelong follow‐up and repeated healthcare encounters. Financial barriers may therefore reduce adherence to routine monitoring and ongoing diabetes management.

#### 4.2.2. Structural Factors

Two subthemes were identified: “lack of infrastructure” and “technical support and regulatory issues.” Poor system integration, inflexible platforms, and lack of technical assistance were reported in more than 11 studies [34, 38, 51, 53, 57, 63, 64, 74, 75, 91, 92]. Regulatory barriers such as restrictive licensing, inconsistent reimbursement policies, and workflow disruptions were noted as systemic impediments [46, 91]. Altabtabaei et al. [34] emphasized that even when patients were willing, digital unpreparedness at the organizational level often undermined service delivery. In addition, Aberer et al. [18] noted that although telemedicine offers viable alternatives to in‐person consultations in outpatient diabetes care and can improve access, its widespread adoption remains hindered by multiple structural barriers including resistance to change, skepticism about time and cost savings, limited availability, and unresolved technical and regulatory challenges.

For patients with diabetes, these structural barriers may interrupt continuous monitoring, delay communication with healthcare professionals, and reduce the effectiveness of long‐term disease management, all of which are essential components of diabetes care.

#### 4.2.3. Communication Factors

This theme encompassed two subthemes: “difficulty communicating and using” and “lack of social support.” Language incompatibility, unclear interfaces, and absence of native‐language support were barriers in multiple studies [34, 47, 54, 80, 92]. Patients also reported feeling less heard or attended to during virtual visits, discouraging open dialogue [38, 53]. Family or peer support was identified as a crucial enabler in rural and elderly populations; its absence significantly reduced engagement [71, 88]. This may be particularly problematic in diabetes care, where individualized counseling, discussion of self‐monitored glucose values, and shared decision‐making are essential components of effective disease management.

#### 4.2.4. Attitudinal Factors

Four subthemes emerged as subsets of this theme: “concerns,” “lack of awareness,” “lack of desire and interest,” and “low quality.” Privacy and data security concerns were cited in seven studies [42, 53, 63, 64, 68, 75, 80]. Low digital literacy and resistance to change were reported in 11 studies, particularly among older adults [1, 17, 34, 41, 44, 72, 74, 80–82, 92]. Many patients remained unaware of available services [74, 81, 88, 89], whereas others preferred in‐person care due to perceived warmth, thoroughness, or diagnostic reliability [1, 38, 57, 82]. Importantly, perceptions of low‐quality care—such as feeling providers were distracted or less engaged—were recurrent [38, 51, 57, 68]. These attitudinal barriers highlight that technology alone is insufficient; human‐centered design and empathetic communication remain essential. Given that diabetes self‐management increasingly relies on digital technologies for glucose monitoring, education, and communication, inadequate digital literacy may disproportionately affect patients′ ability to engage successfully with telehealth services.

## 5. Strengths and Limitations

The primary strength of this study lies in its comprehensive mapping of facilitators and barriers to telehealth adoption in diabetes care, synthesized into a unified, patient‐centered framework. This scoping review adhered rigorously to the JBI and PRISMA‐ScR guidelines, ensuring methodological transparency and reproducibility.

Consistent with current JBI guidance for scoping reviews, a formal critical appraisal of the included studies was not undertaken because the primary objective was to map the breadth and nature of the available evidence rather than evaluate its methodological quality. Consequently, the reported facilitators and barriers should be interpreted as a comprehensive synthesis of the existing literature rather than as evidence weighted according to study quality. Furthermore, the included studies exhibited substantial methodological heterogeneity in terms of study design, setting, sample size, and data collection methods, which may have influenced the consistency and generalizability of the reported findings.

This heterogeneity also precluded direct comparison of findings across studies and prevented assessment of whether specific facilitators or barriers were consistently associated with healthcare systems, geographic regions, or study designs.

Another limitation of this review is that only full‐text articles published in English or Persian were included. As a result, potentially relevant studies published in other languages may have been missed, introducing language bias and limiting the comprehensiveness and, to some extent, the generalizability of the synthesized evidence. Future reviews that include studies published in additional languages may provide a more comprehensive understanding of patient‐reported facilitators and barriers to telehealth consultations.

## 6. Conclusion

Telehealth consultations offer substantial promise for improving diabetes management—a critical need in chronic disease care. Key facilitators such as time and cost savings, enhanced communication, increased patient education, and advancements in virtual system capabilities can collectively reduce healthcare burdens and elevate care quality. Conversely, persistent barriers including infrastructural deficits, technical challenges, cost‐related concerns, and low awareness or motivation among patients and providers continue to hinder widespread adoption.

This review addresses a critical gap in the literature: the absence of a holistic, systematically organized framework capturing the full spectrum of telehealth facilitators and barriers. Its core contribution is the novel integration of both well‐documented and underrecognized factors into four interconnected, patient‐centered domains: “process,” “structural,” “communication,” and “attitudinal” factors. More than a mere inventory, this framework serves as a practical roadmap for clinicians, administrators, and policymakers to design multilevel interventions that simultaneously target logistical, technological, relational, and psychological dimensions—thereby accelerating equitable, effective, and sustainable telehealth integration in diabetes care globally.

### 6.1. Implications for Practice and Research

The findings indicate that telehealth programs for diabetic patients must be designed with patient‐centered principles at their core. Key features should include cost‐effective access, intuitive and user‐friendly interfaces, multilingual functionality, and robust technical support to address disparities in digital literacy. Systems should also prioritize privacy, personalization, and seamless integration with existing care workflows to enhance trust and usability. In resource‐constrained health systems, including many low‐ and middle‐income countries, these findings suggest that telehealth policies should prioritize affordable internet access, integration of telehealth into primary healthcare services, reimbursement mechanisms that reduce out‐of‐pocket costs, and targeted digital literacy support for older adults and socioeconomically disadvantaged patients. Policymakers should also consider multilingual platforms, low‐bandwidth solutions, and hybrid models that combine virtual and in‐person care to accommodate varying levels of technological access and patient preference. Such context‐sensitive strategies may help improve equity, acceptability, and sustainable adoption of telehealth services for people living with diabetes. For future research, it is critical to examine how postpandemic policy shifts—particularly regarding reimbursement, licensure, and data regulation—continue to influence patient preferences and adoption patterns. Additionally, the role of RPM technologies in shaping long‐term engagement and clinical outcomes warrants deeper investigation. Crucially, future studies should not only assess policy impacts from provider or organizational viewpoints but must also explore how these structural changes indirectly affect patient decision‐making, perceived value, and lived experiences with telehealth. This dual lens will ensure that policy development remains aligned with patient needs and real‐world usability.

**Nomenclature**
CASP: Critical Appraisal Skills Program
HCPs: health care providers
ICT: information and communication technology
MHLW: Ministry of Health, Labor, and Welfare
MMAT: Mixed Methods Appraisal Tool
NHS: National Health Service
PRISMA: Preferred Reporting Items for Systematic Reviews and Meta‐Analyses
SANRA: Assessment of Narrative Review Articles
T2D: type 2 diabetes
T2DM: type 2 diabetes mellitus

## Author Contributions

Mahsa Fadaie Fini, Tahereh Shafaghat, Seyyed Reza Darijani, Mahdi Radabadi, and Mohammad Ranjbar contributed to the conception and design of the study. Mahsa Fadaie Fini and Tahereh Shafaghat collected the data. Tahereh Shafaghat, Mohammad Ranjbar, Seyyed Reza Darijani, Enayatollah Homaie Rad, and Sajjad Bahariniya analyzed and interpreted the data. Mahsa Fadaie Fini, Tahereh Shafaghat, and Mohammad Ranjbar wrote the first draft of the manuscript. Tahereh Shafaghat, Sajjad Bahariniya, and Mohammad Ranjbar contributed to the substantial revisions, addressed the reviewers′ comments, and applied the necessary changes after peer review. All authors read and approved the submitted version.

## Funding

No funding was received for this manuscript.

## Disclosure

All authors read and approved the final manuscript.

## Ethics Statement

The study has been approved by the Research Ethics Committee of Shahid Sadoughi University of Medical Sciences, with Reference Number: IR.SSU.SPH. REC.1402.184.

## Conflicts of Interest

The authors declare no conflicts of interest.

## Supporting information


**Supporting Information** Additional supporting information can be found online in the Supporting Information section. The supporting materials provide a comprehensive summary of the characteristics of the included studies, including publication year, country, study title, study design, setting, target population, type of telehealth intervention, and key reported facilitators and barriers.

## Data Availability

All relevant data are included within the manuscript and its supporting information.
