# Supplementary material for: Factors Affecting Telehealth Consultation in Diabetic Patients: A Systematic Scoping Review
Source: Int J Telemed Appl. 2026 Aug 16;2026:4696370. doi: 10.1155/ijta/4696370 (PMC13478728; doi:10.1155/ijta/4696370)
Supplement: Supplementary file 1 [file IJTA-2026-4696370-s001.docx]

Supplementary table. Summary of Characteristics of Included Studies

| No. | First Author (Year) | Country | Study Title | Study Design | Setting | Participant Demographics | Type of Telehealth Intervention | Key Findings (Telehealth Facilitators (F) & Barriers (B)) |
| --- | --- | --- | --- | --- | --- | --- | --- | --- |
| 1 | Armstrong, N (2007) | UK | Design of a trial of Internet-based self-management for diabetes | Qualitative study | Not specified | Patients with diabetes | Internet-based self-management | F: Patient involvement in design |
| 2 | Östlund, M. (2011) | Sweden | 3D visualization as a communicative aid in pharmaceutical advice-giving over distance | Mixed-method | Pharmacy call center; General public | 1st study: 10 students, 2 consultants. 2nd study: 23 public, 3 consultants. | Video consultation with 3D visualization tool | F: Improved communication via visual aids.  B: Lack of familiarity with digital technologies |
| 3 | Chisolm D. J. (2011) | USA | What makes teens start using and keep using health information web sites? a mixed model analysis of teens with chronic illnesses | Qualitative study (semistructured telephone interview) | asthma and diabetes specialty clinics | 180 teens, aged 13-18, were recruited.  129 participants completed a 3-month follow-up | health information websites | F: Perceived usefulness, ease of use  B: Highlights perceived limitation of telemedicine (lack of physical exam) |
| 4 | Basoglu, N. (2012) | Turkey | Determining Patient Preferences for Remote Monitoring | Quantitative study | Not specified | Patients with diabetes and obesity | Remote monitoring | F: Preferences for specific features |
| 5 | Kouroubali, A. (2012) | 8 European countries | Developing advanced technology services for diabetes management: User preferences in Europe | Qualitative study (focus groups) | Not specified | Focus groups (6-8 people each) | Advanced technology services | F: User preferences for features |
| 6 | Kurji, K. (2013) | Kenya | Improving diabetic retinopathy screening in Africa: patient satisfaction with tele-ophthalmology versus ophthalmologist-based screening | structured questionnaire | Diabetic retinopathy screening | Diabetic patients (n=57) | Tele-ophthalmology | F: High satisfaction with tele-ophthalmology |
| 7 | Fatehi F. (2014) | Australia | Success Factors and Challenges for Establishing the Princess Alexandra Hospital Tele- Endocrinology Clinic in Brisbane, Australia: A Qualitative Study | Qualitative Study (semistructured interview) | Hospital diabetes & endocrinology dept. | Head of dept., endocrinologist, diabetes nurses (n=5) | Telemedicine clinic setup | F/B: Technical factors, organizational-managerial factors, cultural and sociological factors, financial and economic factors, and factors related to ethical considerations |
| 8 | Saddik, B. (2015) | Saudi Arabia | Diabetic patients' willingness to use tele-technology to manage their disease - A descriptive study | Quantitative (Cross-sectional exploratory study) | Not specified | Participants (n=102) | Tele-technology for management | F: Willingness to use  B: Lack of awareness |
| 9 | Keely E. (2015) | Canada | Patients' Perspectives on Wait Times and the Referral-Consultation Process While Attending a Tertiary Diabetes and Endocrinology Centre: Is E-consultation an Acceptable Option? | 2-stage patient survey and a chart audit | Tertiary Diabetes and Endocrinology Centre | Patients with diabetes and endocrine disorders (n=101) | E-consultation | F: Acceptability as an option |
| 10 | Samiei, V. (2016) | Malaysia | Are Malaysian Diabetic Patients Ready to Use The New Generation of Health Care Service Delivery? A Telehealth Interest Assessment | Quantitative (Cross-sectional study) | Not specified | Patients with type 2 diabetes (n=180) | Telehealth services (conceptual) | F: Interest in future use  B: Low awareness, need for education |
| 11 | Ramirez, M. (2016) | USA | Automated Remote Monitoring of Depression: Acceptance Among Low-Income Patients in Diabetes Disease Management | Quantitative (longitudinal) | Disease management program | Low-income patients with diabetes | Automated remote depression monitoring | F: Acceptability among low-income groups |
| 12 | Peng, W. (2016) | USA | Exploring the Challenges and Opportunities of Health Mobile Apps for Individuals with Type 2 Diabetes Living in Rural Communities | Qualitative study (Interview) | Rural communities | Individuals with T2D (n=18) | Health mobile apps | F: Free apps, tangible rewards |
| 13 | Valikodath, N. G. (2017) | USA | Patient Attitudes Toward Telemedicine for Diabetic Retinopathy | Survey | Diabetic Retinopathy screening | Participants (n=97) | Telemedicine for Diabetic Retinopathy | F: Positive attitudes |
| 14 | Muigg, D. (2018) | Austria | Is Austria ready for Telemonitoring? A readiness assessment among doctors and patients in the field of diabetes | Cross-sectional Survey | Diabetes field | Physicians (n=41), Patients (n=47), Stakeholder (n=28) | Telemonitoring | F/B: Assessed readiness levels among groups |
| 15 | Xu, T. (2018) | USA | Telemedicine in the Management of Type 1 Diabetes. Preventing chronic disease | retrospective chart review | Atlanta Endocrinology Telehealth Clinic | 32 patients with type 1 diabetes | Telehealth | F: time savings, cost savings, high appointment  adherence rates, and high patient satisfaction |
| 16 | Adu, M. D. (2018) | Australia, Europe, Asia and America | Users' preferences and design recommendations to promote engagements with mobile apps for diabetes self-management: Multi-national perspectives | Mix-method | Multi-national | Patients with type 1 diabetes (n=217) | Mobile apps for self-management | F/B: Gathered preferences and design recommendations  B: Lack of internet, high cost, lack of awareness/interest |
| 17 | Jemere, A. T. (2019) | Ethiopia | Access to mobile phone and willingness to receive m-Health services among patients with diabetes in Northwest Ethiopia: a cross-sectional study | Cross-sectional survey | Diabetes clinic | Patients with diabetes (n=423) | m-Health services (conceptual) | F: Mobile phone ownership. B: Lack of access to phones/internet, cost |
| 18 | Rogers, E. (2019) | USA | Barriers and Facilitators to the Implementation of a Mobile Insulin Titration Intervention for Patients With Uncontrolled Diabetes: A Qualitative Analysis | Qualitative (depth interviews) | MITI program | Patients (n=36), Staff (n=19) | Mobile Insulin Titration Intervention (MITI) | F: Characteristics of intervention, patient/staff attitudes  B: Internal settings, implementation processes |
| 19 | Iftikhar, S. (2019) | Pakistan | Capacity and willingness to use information technology for managing chronic diseases among patients: A cross-sectional study in Lahore, Pakistan | Descriptive, cross-sectional | Not specified | Patients with chronic diseases (n=400) | IT for chronic disease management | F: Capacity to use  B: Willingness low due to cost, lack of awareness |
| 20 | Muigg D. (2019) | Austria | Telemonitoring readiness among Austrian diabetic patients: A cross-sectional validation study | Cross-sectional Study | Not specified | Diabetic patients (n=41) | Telemonitoring | F/B: Assessed readiness levels |
| 21 | Rodríguez-Fortúnez, P. (2019) | Spain | Cross-sectional study about the use of telemedicine for type 2 diabetes mellitus management in Spain: patient's perspective. The FnRFDa2 Study | Observational, cross-sectional | Not specified | Patients with T2DM (n=1036) | Telemedicine for management | F: Interest in use  B: Age, lower education, lack of information |
| 22 | Holubová, A. (2019) | Kenya | Customizing the Types of Technologies Used by Patients With Type 1 Diabetes Mellitus for Diabetes Treatment: Case Series on Patient Experience | Qualitative study (case series) | Not specified | Patients with T1DM (n=6; 3M, 3F) | Various technologies for treatment | F: Customization to patient needs |
| 23 | Haluza, D. (2019) | UK, and Austria | Future of information technology and telecommunication in type 1 diabetes clinical care: Results of an online survey | Survey (Cross-sectional) | Online | Patients with T1D (n=294) | IT and telecommunication in care | F: Positive outlook on future use |
| 24 | Rafiullah, M. (2019) | Saudi Arabia | Health apps usage and preferences among Saudi patients with diabetes: A survey | Cross-sectional Survey | Not specified | Patients with diabetes (n=399) | Health apps | F: Usage and preferences |
| 25 | Harrison, T.N. (2020) | USA | Acceptability of Virtual Prenatal Visits for Women with Gestational Diabetes | Mixed method (Surveys & qualitative interviews) | Kaiser Permanente | Women with Gestational Diabetes (GDM) (n=70) | Virtual prenatal visits | F: Convenience, time-saving  B: Preference for in-person care for new issues |
| 26 | Jeganathan, S. (2020) | USA | Adherence and acceptability of telehealth appointments for high-risk obstetrical patients during the coronavirus disease 2019 pandemic | Qualitative study (Surveys) | High-risk obstetric clinic | High-risk obstetrical patients & their providers | Telehealth visits (video/phone) | F: Improved access to care, high satisfaction |
| 28 | Sin, D. Y. E. (2020) | Singapore | Assessment of willingness to Tele-monitoring interventions in patients with type 2 diabetes and/or hypertension in the public primary healthcare setting | Cross-sectional survey | Public primary care | Patients with T2D/hypertension (n=899) | Tele-monitoring | F: Willingness to use  B: Age, ethnicity, income, privacy concerns |
| 29 | Kadoya, Y. (2020) | Japan | Disease Control Status and Safety of Telemedicine in Patients With Lifestyle Diseases　- A Multicenter Prospective Observational Study in Japan | Multicenter prospective observational study | Multicenter | Patients with lifestyle diseases (n=34; inc. diabetes, hypertension, dyslipidemia) | Telemedicine | F: Safety and efficacy |
| 30 | Fung, A. (2020) | Canada | Evaluation of telephone and virtual visits for routine pediatric diabetes care during the COVID-19 pandemic | Quantitative study (cross-sectional) | Pediatric diabetes care | Participants (n=87) | Telephone and virtual visits | F: High usability, interest in future use (72%) |
| 31 | Gordon, H. S. (2020) | USA | "I'm Not Feeling Like I'm Part of the Conversation" Patients' Perspectives on Communicating in Clinical Video Telehealth Visits | Qualitative study (semi-structured Interview) | Not specified | Patients with diabetes (n=27) | Clinical Video Telehealth Visits | B: Perception of less attention from provider, difficulty speaking/asking questions |
| 32 | Steinman L (2020) | Cambodia | Facilitators and Barriers to Chronic Disease Self-Management and Mobile Health Interventions for People Living With Diabetes and Hypertension in Cambodia: Qualitative Study | Qualitative Study | Cambodia | Patients with diabetes and hypertension (n=59) | Mobile Health Interventions | B: Lack of family support, gender inequalities |
| 33 | Ju, C. (2020) | China | Influencing Factors of Continuous Use of Web-Based Diagnosis and Treatment by Patients With Diabetes: Model Development and Data Analysis | Correlation factor and Regression analysis | Web-based platform | Data records (n=2437) | Web-Based Diagnosis and Treatment | F: Factors influencing continuous use |
| 34 | Abbasi, R. (2020) | Iran | Investigating the attitude of patients with chronic diseases about using mobile health | Descriptive-Analytical | Not specified | Patients with chronic diseases (n=222; diabetes, asthma, MS) | Mobile health | F: Generally positive attitude |
| 35 | Olamoyegun, M. A. (2020) | Nigeria | Mobile phone ownership and willingness to receive m-Health services among patients with diabetes mellitus in South-West, Nigeria | Cross-sectional Descriptive Study | South-West Nigeria | Patients with diabetes (n=259) | m-Health services | F: Phone ownership, willingness  B: Lack of access, awareness |
| 36 | Ramchandran, R. S. (2020) | USA | Patient perceived value of tele-ophthalmology in an urban, low income US population with diabetes | Mixed Methods Approach | Urban, low income | Patients with T2D (n=23) | Tele-ophthalmology | F: Perceived value in access |
| 37 | Sommer, J. (2020) | Germany | Preferences of people with type 2 diabetes for telemedical lifestyle programs in Germany: protocol of a discrete choice experiment | Discrete Choice Experiment | Not specified | People with T2DM (n=850) | Telemedical lifestyle programs | F/B: Protocol for assessing preferences |
| 38 | Commissariat P. V. (2020) | USA | That's How You Connect with Someone": Preferences of Minority Young Adults (YA) with Type 1 Diabetes (T1D) for In-Person Visits over Telemedicine | Qualitative Study (semi-structured interviews) | Minority youth | Minority youth with T1D | Telemedicine | B: Strong preference for in-person visits; high cost, lack of time |
| 39 | H. Abduo (2020) | Kuwait | Use of Smartphone Applications for Diabetes Management in Kuwait: A Pilot Study | Cross-sectional pilot study | Not specified | Patients with diabetes (n=50; Mean age: 50.6 years; 58% female) | Smartphone applications for self-management (e.g., blood glucose tracking, diet monitoring) | F: Ease of use, perceived usefulness for daily management  B: Lack of awareness of available apps, low digital literacy among older patients, preference for traditional methods, concerns about data accuracy |
| 40 | Aberer, F. (2021) | Austria | Application of Telemedicine in Diabetes Care: The Time is Now | Review article | N/A | N/A (Review of 47 studies) | Various telemedicine interventions | F: Effective alternative  B: Reluctance to adopt, doubts about cost-effectiveness, technical/regulatory concerns |
| 41 | Islam, M. S. (2021) | Bangladesh | Community engagement in the telehealth service for aged people with diabetes: covid-19 response in Bangladesh | Mixed-method | Community setting | Aged people with diabetes (n=100) | Telehealth service | F: Community support  B: Lack of family support, poor counseling, gender inequalities |
| 42 | Luo, J. (2021) | USA | Evaluating the Potential Use of Smartphone Apps for Diabetes Self-Management in an Underserved Population: A Qualitative Approach | Qualitative study (Interview) | Underserved population | Participants (n=15) | Smartphone apps for self-management | F: Potential for self-management  B: Digital literacy, access |
| 43 | Schiller, T. (2021) | Israel | Following the COVID-19 Experience, Many Patients with Type 1 Diabetes Wish to Use Telemedicine in a Hybrid Format | structured questionnaire | Not specified | Patients with T1D (n=126) | Telemedicine | F: Desire for hybrid model post-COVID |
| 44 | March, C. A. (2021) | USA | How Do Virtual Visits Compare? Parent Satisfaction With Pediatric Diabetes Telehealth During the COVID-19 Pandemic | Cross-sectional Survey | Pediatric diabetes | Parents of child with diabetes (n=12) | Pediatric Diabetes Telehealth | F: High satisfaction |
| 45 | Sim, R. (2021) | Singapore | Patient Preference and Satisfaction with the Use of Telemedicine for Glycemic Control in Patients with Type 2 Diabetes: A Review | Review | N/A | N/A (Review of 20 articles) | Telemedicine for glycemic control | F/B: Synthesized preferences and satisfaction factors |
| 46 | Rego, N. (2021) | Portugal | Perceptions of Patients and Physicians on Teleconsultation at Home for Diabetes Mellitus: Survey Study | Survey Study | Not specified | Patients (n=75), Physicians (n=68) | Teleconsultation at home | F/B: Compared perceptions of patients and physicians |
| 47 | Lee, P. S. (2021) | Scotland | The value of physical examination in the era of telemedicine | Not specified | Not specified | Not specified | Telemedicine | B: Highlights perceived limitation of telemedicine (lack of physical exam) |
| 48 | Poitras, M. E. (2022) | Canada | Chronic conditions patient's perception of post-COVID-19 pandemic teleconsulting continuation in primary care clinics: a qualitative descriptive study | Qualitative descriptive study | Primary care clinics | Patients with chronic conditions (n=39) | Teleconsulting (phone) | F: Time-saving, avoided travel  B: Suitability only for certain conditions, relationship with provider |
| 49 | Greenfield, M. (2022) | USA | Diabetes Education and Support Tele-Visit Needs Differ in Duration, Content, and Satisfaction in Older Versus Younger Adults | Cross-sectional study | Not specified | Participants (n=982) | Diabetes education tele-visits | F: Different needs met  B: Age-related differences in satisfaction |
| 50 | Bin Zaman, S. (2022) | Australia | Exploring Barriers to and Enablers of the Adoption of Information and Communication Technology for the Care of Older Adults With Chronic Diseases: Scoping Review | Scoping Review | N/A | Participants (n=33; 23 patients, 10 providers) | ICT for care | F: Non-pharmacological, health education, fun  B: Knowledge gaps, reluctance to adopt new skills, unwillingness |
| 51 | Vicente, M. A. (2022) | Spain | Patient Engagement Using Telemedicine in Primary Care during COVID-19 Pandemic: A Trial Study | Trial Study | Primary care | Patients with chronic conditions (n=70) | Telemedicine | F: Improved patient engagement |
| 52 | Sanmarchi, F. (2022) | Australia | Perceived quality and willingness to continue using telemedicine services in patients with diabetes | Structured Survey | Not specified | Patients with diabetes (n=569) | Telemedicine services | F: Willingness to continue linked to perceived quality |
| 53 | Cheng, K. (2022) | China | Perceptions of Chinese older adults with type 2 diabetes mellitus about self-management mobile platform: A qualitative study | Qualitative study (semi-structured interview) | Not specified | Older adults with T2DM (n=20) | Self-management mobile platform | F/B: Explored perceptions and potential barriers |
| 54 | Chua, V. J, (2022) | Singapore | The Willingness to Pay for Telemedicine Among Patients With Chronic Diseases: Systematic Review | Systematic literature review | N/A | N/A (Review of 11 studies) | Telemedicine | F/B: Synthesized evidence on willingness to pay |
| 55 | Sun, C. A. (2023) | USA | Experiences and Perceptions of Telehealth Visits in Diabetes Care During and After the COVID-19 Pandemic Among Adults With Type 2 Diabetes and Their Providers: Qualitative Study | Qualitative study | Not specified | Adults with T2D (n=23) & their providers | Telehealth visits | F: Perceived benefits (convenience).  B: Perceived challenges (tech issues), impact on quality of care |
| 56 | Altabtabaei, R. (2023) | Kuwait | Exploring the Challenges and Opportunities of Adopting and Using Telemedicine for Diabetes Care and Management: Qualitative Semi structured Interview Study Among Health Care Providers and Patients With Diabetes | Qualitative and Exploratory (semi-structured interviews) | Not specified | Participants (n=20; 10 HCPs, 10 patients) | Telemedicine for care & management | F: User-friendly system, safety during COVID  B: Lack of system integration, language barriers, poor internet |
| 57 | Sze, W. T. (2023) | Malaysia | Perspectives and Needs of Malaysian Patients With Diabetes for a Mobile Health App Support on Self-Management of Diabetes: Qualitative Study | Qualitative Study (semi-structured online interviews) | Not specified | Participants (n=15) | Mobile Health App | F/B: Identified perspectives and needs for app design |
| 58 | Walle, A. D. (2023) | Ethiopia | Willingness of diabetes mellitus patients to use m-Health applications and its associated factors for self-care management in a low-income country: an input for digital health implementation | Cross-Sectional Study | Ethiopia | Patients with diabetes (n=422) | m-Health applications | F: Willingness to use  B: Associated factors (age, education, etc.) |
| 59 | Mukherjee, S.M. (2023) | USA | Pharmacist telehealth in an underserved urban population with type 2 diabetes mellitus | Prospective Cohort | Underserved urban clinic | Underserved urban adults with T2DM (n=71); Mean age: 56 years; 62% female | Pharmacist-led telehealth (Telephone consultations for medication management) | F: Cost-saving, Improved communication, Quality of care |
| 60 | Fan, J.X. (2023) | China | Health economic evaluation of telemedicine diabetic retinopathy examination using a non-mydriatic fundus camera in China | Economic Evaluation | Healthcare system | Patients with diabetes; Demographic details not fully specified | Tele-ophthalmology (Retinal imaging + remote diagnosis) | F: Cost-saving, Quality/efficacy |
| 61 | Cox, E.R. (2024) | Australia | Feasibility, safety and preliminary efficacy of telehealth-delivered group exercise for people with type 2 diabetes: A pilot trial | Pilot Trial | Community setting | Adults with T2DM (n=25); Mean age: 60.4 ± 9.7 years; 48% female | Telehealth-delivered group exercise (Supervised group sessions via video conference) | F: Quality/efficacy, Ease of use  B: Technological barriers (internet access) |
| 62 | Cyrino, F.V.R. (2024) | Brazil | Applicability of portable retinal cameras and telemedicine as facilitating tools in screening diabetic retinopathy in the COVID-19 pandemic scenario | Diagnostic Study | Screening clinics | Patients with diabetes; Demographic details not fully specified | Tele-ophthalmology (Retinal imaging with portable cameras + remote interpretation) | F: Quality/efficacy (access to screening), Structural capabilities. |
| 63 | Hong, Y.R. (2024) | USA | Telehealth service use and quality of care among US adults with diabetes: A cross-sectional study of the 2022 health information national trends survey | Cross-sectional Survey | National survey | US adults with diabetes (n=1,201); Age: ≥18 years (categorized) | General Telehealth Use (Video, phone, messaging, etc.) | F/B: Cost (insurance coverage), Privacy concerns, Attitudinal factors (perceived quality) |
| 64 | Lee, J.M. (2024) | USA | Institutional Barriers to the Successful Implementation of Telemedicine for Type 1 Diabetes Care | Qualitative | Healthcare systems | Healthcare professionals and leaders involved in T1D care | Telemedicine implementation (Focus on system-level integration) | B: Structural factors (Workflow integration, reimbursement, technical support) |
| 65 | Rodriguez, H.P. (2024) | USA | Telemedicine use and decrements to type 2 diabetes and hypertension care during the COVID-19 pandemic | Mixed-Methods | Healthcare systems | Patients with T2DM or hypertension; Demographic details from larger cohort | Telemedicine use (Analysis of visit types and care processes) | B: Structural factors (Care delays, workflow challenges) |
| 66 | Rodríguez, S.M.A. (2024) | Colombia | Telehealth in the medical care of type 2 diabetic patients: Clinical outcomes, advantages, and disadvantages | Observational | Clinical setting | Patients with T2DM; Age and sex not specified | General Telehealth (Remote consultations and monitoring) | F: Process factors (time/cost savings)  B: Communication difficulties |
| 67 | Singhal, M. (2024) | New Zealand | Telehealth and Technology for Diabetes in Pregnancy Clinics: Staff Perspectives from South Auckland, New Zealand | Qualitative | Diabetes-in-pregnancy clinics | Healthcare staff (n=16) in diabetes-in-pregnancy clinics | Telehealth in specialist clinics (Views on various remote technologies) | B: Communication factors (Language barriers, low digital literacy), Technical issues |
| 68 | Yip, O. (2024) | USA | Comparison between in-person, telehealth, and combination visits among veterans treated in a pharmacist-led diabetes management clinic | Retrospective Cohort | VA healthcare system | Veterans with diabetes (n=278); Mean age: 66.3 years; 96.8% male. High comorbidity rate | Pharmacist-led telehealth (Telephone and video consultations) | F: Process factors, perceived convenience |
| 69 | Shih, J.J. (2025) | USA | Differences in Telemedicine Use for Patients With Diabetes in an Academic Versus Safety Net Health System: Retrospective Cohort Study | Retrospective Cohort | Academic vs. Safety-Net health systems | Patients with diabetes from Academic (n=16,803) vs. Safety-Net (n=5,163) health systems. Demographics differed significantly | Telemedicine visits (Video and telephone) | B: Structural factors (limited technology access), Attitudinal factors (preference for in-person) |
| 70 | Lyons, J. (2025) | USA | Pandemic Telehealth Utilization Among Ambulatory Care Diabetes Patients Managed at an Urban Teaching Hospital in Washington, DC | Retrospective Cohort | Urban teaching hospital | Ambulatory care patients with diabetes (n=5,163); Mean age: 63.4 years; 57.4% female, 77.4% Black. High comorbidity burden | Telehealth visits (Video and telephone visits) | F: Process factors (accessibility)  B: digital literacy |
| 71 | Chichkova, V.V.  (2025) | Russia | Application of telemedicine technologies in therapeutic education of patients with type 2 diabetes mellitus | Interventional | Clinical setting | Patients with T2DM; Age and sex not specified | Remote therapeutic education (Structured educational programs) | F: Patient education and awareness, Quality of service delivery |
| 72 | Jewell, V.D. (2025) | USA | Rural Caregiver Perceptions on the Content and Receipt of a Diabetes Management Telehealth Occupation-Based Coaching Intervention | Qualitative | Rural communities | Rural caregivers of individuals with diabetes (n=7); Mostly female | Telehealth coaching (Occupation-based coaching via video call) | F: Improved communication/access  B: Infrastructure limitations (internet access) |
